# Secondary cancer‐incidence risk estimates for external radiotherapy and high‐dose‐rate brachytherapy in cervical cancer: phantom study

**DOI:** 10.1120/jacmp.v17i5.6087

**Published:** 2016-09-08

**Authors:** Boram Lee, Sung Hwan Ahn, Hyeyoung Kim, Jaeman Son, Jiwon Sung, Youngyih Han, Seung Jae Huh, Jin Sung Kim, Dong Wook Kim, Myonggeun Yoon

**Affiliations:** ^1^ Department of Bio‐convergence Engineering Korea University Seoul; ^2^ Department of Radiation Oncology Samsung Medical Center Seoul; ^3^ Department of Radiation Oncology Samsung Medical Center, Sungkyunkwan University School of Medicine Seoul; ^4^ Department of Radiation Oncology College of Medicine, Yonsei Cancer Center Seoul; ^5^ Department of Radiation Oncology Kyung Hee University Hospital at Gangdong Seoul Korea

**Keywords:** secondary cancer risk, brachytherapy, cervical cancer, lifetime attributable risks

## Abstract

This study was designed to estimate radiation‐induced secondary cancer risks from high‐dose‐rate (HDR) brachytherapy and external radiotherapy for patients with cervical cancer based on measurements of doses absorbed by various organs. Organ doses from HDR brachytherapy and external radiotherapy were measured using glass rod dosimeters. Doses to out‐of‐field organs were measured at various locations inside an anthropomorphic phantom. Brachytherapy‐associated organ doses were measured using a specialized phantom that enabled applicator insertion, with the pelvis portion of the existing anthropomorphic phantom replaced by this new phantom. Measured organ doses were used to calculate secondary cancer risk based on Biological Effects of Ionizing Radiation (BEIR) VII models. In both treatment modalities, organ doses per prescribed dose (PD) mostly depended on the distance between organs. The locations showing the highest and lowest doses were the right kidney (external radiotherapy: 215.2 mGy; brachytherapy: 655.17 mGy) and the brain (external radiotherapy: 15.82 mGy; brachytherapy: 2.49 mGy), respectively. Organ doses to nearby regions were higher for brachytherapy than for external beam therapy, whereas organ doses to distant regions were higher for external beam therapy. Organ doses to distant treatment regions in external radiotherapy were due primarily to out‐of‐field radiation resulting from scattering and leakage in the gantry head. For brachytherapy, the highest estimated lifetime attributable risk per 100,000 population was to the stomach (88.6), whereas the lowest risks were to the brain (0.4) and eye (0.4); for external radiotherapy, the highest and lowest risks were to the thyroid (305.1) and brain (2.4). These results may help provide a database on the impact of radiotherapy‐induced secondary cancer incidence during cervical cancer treatment, as well as suggest further research on strategies to counteract the risks of radiotherapy‐associated secondary malignancies.

PACS number(s): 87.52.‐g, 87.52.Px, 87.53.Dq, 87.53.Jw

## I. INTRODUCTION

Cervical cancer, which primarily affects middle‐aged women, is one of the most common gynecologic malignancies, along with breast cancer. The standard of care for cervical cancer has progressed from external beam radiation therapy (EBRT) alone, to EBRT plus brachytherapy.^1^, ^2^, ^3^ EBRT, administered at a dose appropriate for controlling microscopic disease, is generally performed to treat pelvic lymph nodes, parametria, and the primary tumor, whereas brachytherapy is administered to treat the gross tumor, improving disease control with better survival.^4^, ^5^, ^6^, ^7^, ^8^ Recent advances in brachytherapy have resulted in increased replacement of the conventional two‐dimensional (2D) treatment system by a three‐dimensional (3D) treatment system. Outcome analysis in the context of radiation therapy indicated that delineation of gross tumor volume and determination of target volume should be based on various image‐acquisition techniques, including computed tomography (CT), magnetic resonance imaging (MRI), and ultrasonography.^(9)^

The risk of secondary malignancies associated with radiotherapy for cancer patients has become increasingly important, since the mean age of patients at the onset of cancer has been decreasing and the survival rate has been increasing. For example, the five‐year survival rate for all cancer patients in the United States has significantly increased in the past several decades. A recent study assessing the risk of secondary cancer among 647,672 cancer patients treated with and without radiation therapy showed that 60,271 patients developed a second solid cancer, of which 3,266 were considered related to radiotherapy.^(10)^ Similarly, the relative risks of developing secondary cancer for patients surviving >5 and >10 years after EBRT for prostate cancer were estimated to be 15% and 34%, respectively.^(11)^ In comparison, the relative risk of prostate cancer patients developing secondary cancer after brachytherapy was reported to be over 10%. Although various methods have been proposed for the estimation of radiation‐induced cancer risks, these risks remain difficult to estimate and have large uncertainties. Considering stochastic effects of, for example, cancer and heritable disease, the International Commission on Radiological Protection (ICRP) recommended that radiation hazards to organs be estimated using tissue weighting factors and nominal risk coefficients for doses lower than 100 mSv.^(12)^ The Biological Effects of Ionizing Radiation (BEIR) VII committee has developed more specific risk models, using parameters such as sex, organs, exposure, and attained age, to estimate low‐dose exposures of several organs.^13^, ^14^ Although various nonlinear risk models have estimated secondary cancer risks after radiotherapy, the select BEIR VII model for risk estimation is considered reasonable, because doses absorbed by out‐of‐field organs are usually low.

Although there have been many studies of secondary cancer risk after EBRT, fewer have analyzed secondary cancer risk after brachytherapy.^15^, ^16^, ^17^, ^18^, ^19^ For example, although cervical cancer is usually treated with high‐dose‐rate (HDR) brachytherapy, few studies have analyzed secondary cancer risk due to HDR brachytherapy in patients with cervical cancer. This study, therefore, compared radiation‐induced secondary cancer risks from HDR brachytherapy and EBRT for cervical cancer, by measuring the doses of radiation absorbed by various organs.

## II. MATERIALS AND METHODS

### A. Measurement of organ doses using glass dosimeter in an anthropomorphic phantom

**Figure 1:**
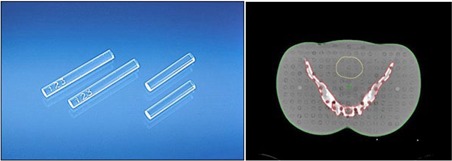
Glass rod dosimeters (left) were inserted into holes of an anthropomorphic phantom (right) to measure organ doses.

Doses to organs were measured using a glass rod dosimeter (GD‐302M) and an automatic readout system (FDG‐1000, Asahi Techno Glass Corporation, Shizuoka, Japan) (Fig. 1).^(20)^ Although TLD is still the major dosimeter for monitoring personal doses, it has the drawback of nonrepeatable readouts for measurements, in contrast to a glass dosimeter.^(21)^ The glass rod dosimeter was 1.5 mm in diameter and 12.0 mm in length, was composed of the chemical elements P (31.55%), O (51.16%), Na (11.00%), Al (6.12%), and Ag (0.17%), had an effective atomic number of 12.04, and a density of $2.61\;g/{\text{cm}}^{3}$. The dosimeter emitted a light signal proportional to the absorbed radiation dose. To prevent scratches or contamination and to ensure easy handling or usage, the glass rod dosimeter was placed in a plastic capsule of length 13 mm, inner diameter 1.8 mm, and outer diameter 2.8 mm, during use.

To prevent confusion among dosimeters, all dosimeters were sequentially numbered. Once the measurement was completed, each dosimeter was preheated at 70°C for 40 min, and the data were collected with the FDG‐1000 readout system. Illumination of the dosimeter with ultraviolet (UV) light at a wavelength of 337.1 nm causes the dosimeter to emit orange luminescence; this visible light signal was amplified by a photomultiplier tube and converted to digital dose data. The glass dosimeters were calibrated using a linear accelerator. That is, the dosimeters were irradiated to determine the sensitivity correction coefficient at the reference condition of SSD 100 cm and a square field of 10 cm. After each readout, the glass rod dosimeter was annealed at 400°C for 1 hr, followed by a gradual cooldown for the next measurement.^(22)^ The glass rod dosimeter was inserted into a female anthropomorphic phantom (Radiology Support Devices, Long Beach, CA).^20^, ^22^, ^23^, ^24^ Four trials were performed with the phantom for each treatment modality. Table 1 shows the organs selected for dose measurements.

**Table 1:** Numbers of glass dosimeters in the phantom and slice number.

*Organ*	*Number*	*PLD ID*	*Phantom Level*
Brain	2	1∼2	3
Eye	2	3∼4	4
Thyroid	2	5∼6	9
Breast	2	7∼8	15
Lung	6	9∼14	13,15,17
Esophagus	2	15∼16	9,16
Liver	3	17∼19	20,21
Left kidney	2	21∼21	21
Right kidney	2	22∼23	22
Stomach	2	24∼25	18,20

### B. Secondary cancer risk calculation model

The excess absolute risk (EAR) and the excess relative risk (ERR) of secondary cancer were calculated using Eq. (1) below, and the lifetime attributable risks (LARs) were evaluated based on two calculated EAR and ERR.^25^, ^26^, ^27^

(1) $$\text{EAR}(D,s,e,a)\;and\;ERR(D,s,e,a)=\beta_{s}dexp(\gamma e^{ast}){\left(\frac{a}{60}\right)}^{\eta }$$

The biological parameters used in Eq. (1) were based on the data obtained from the Hiroshima bombing and Chernobyl accident.^(14)^ D,e, and *a* denote dose, age at exposure, and age attained, respectively. $\beta_{S},\gamma$, and η denote the specific parameters for EAR and ERR. Age attained (*a*) was defined as e + L, with L being a latency period of five years for solid cancers. LAR was calculated from EAR and ERR using Eq. (2).^(13)^

(2) $$LAR_{\left(D,e\right)}={\left(\sum_{a}^{90}ERR(D,e,a)\times \lambda_{1}^{C}\times \frac{S(a)}{S(e)da}\right)}^{0.7}\times {\left(\sum_{a}^{90}ERR(D,e,a)\times \frac{S(a)}{S(e)da}\right)}^{0.3}$$

where $\lambda^{C}$ represents the baseline cancer risk and S(a)/S(e) is the probability of a person of surviving to age a following exposure at age e. Baseline cancer prevalence and survival data were based on relevant statistics for the Korean population. In accordance with the recommendation of the BEIR VII Committee, the weights for EAR and ERR were defined as 0.7 and 0.3, except for certain organs. Only EAR was used to calculate LAR for the breast, based on recommendations to use the EAR model for the breast.^(28)^ Conversely, only ERR was used to calculated LAR for the thyroid, based on the BEIR VII report, which recommended that the EAR model not be used for this organ.^(13)^ Mean age of cervical cancer patients was calculated for 10 randomly selected patients recently treated for cervical cancer; these patients ranged in age from 42–64 years, with a mean age of 53.8 years. Age at exposure was set at 30 years and age attained at 90 years, based on the current life expectancy at birth of Korean females of 85 years.

### C. Dose measurement of external therapy and brachytherapy

**Figure 2:**
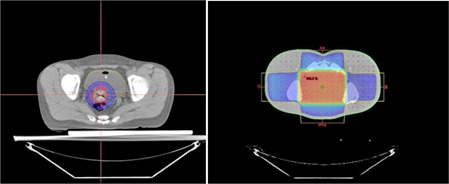
Dose distribution of brachytherapy (left) and external radiation therapy (right).

Our center uses external radiotherapy and brachytherapy to treat patients with cervical cancer. The dose distribution of brachytherapy is more localized than that of external radiotherapy (Fig. 2). The prescribed dose of external radiotherapy in our institution is 1.8 Gy per fraction in 25 fractions delivered to the whole pelvis in the prone position, whereas the planned brachytherapy dose is 4 Gy per fraction in 6 fractions at the same time or after external radiotherapy.

**Figure 3:**
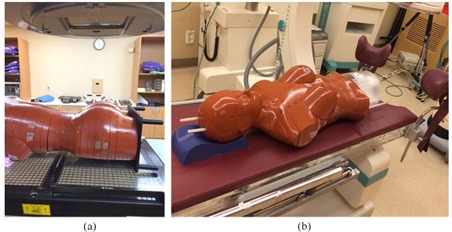
Experimental setup, with anthropomorphic phantoms for external radiotherapy (a) and brachytherapy (b).

The choice of postoperative radiation therapy, whether brachytherapy or external radiotherapy, was based on lymph node involvement or the presence of positive resection margins. The parameters of the 3D treatment planning system were used for evaluation purposes, as 3D planning is the more commonly used system and involves larger organ doses than 2D planning. Both radiotherapy modalities followed the standard protocols used in our center. External radiotherapy used the four‐field box technique, Pinnacle v9.8 (Philips, Eindhoven, Netherlands) as a treatment planning tool and 10 MV photon energy beams from the Varian 21EX (Varian Medical Systems, Palo Alto, CA). While performing the brachytherapy experiment, a problem related to dosimetry was noted. The applicator (tandem and ovoid) necessary for brachytherapy could not be inserted in the existing anthropomorphic phantom. A specialized phantom of the pelvic portion enabling the insertion of the applicator was fabricated from acrylic and used to replace the corresponding part of the existing phantom. Figures 3(a) and 3(b) show the phantom‐based experimental setups for external radiotherapy and brachytherapy, respectively. Applicators for brachytherapy consisted of a 30° intrauterine and 20 mm small ovoid pair (Fletcher Williamson Applicator Set Part No. 080.230; Nucletron BV, Veenendaal, Netherlands). 3D treatment planning with a ^192^Ir source was performed using Oncentra (Nucletron BV) and therapy administered with MicroSelectron (Nucletron BV).

## III. RESULTS

**Figure 4:**
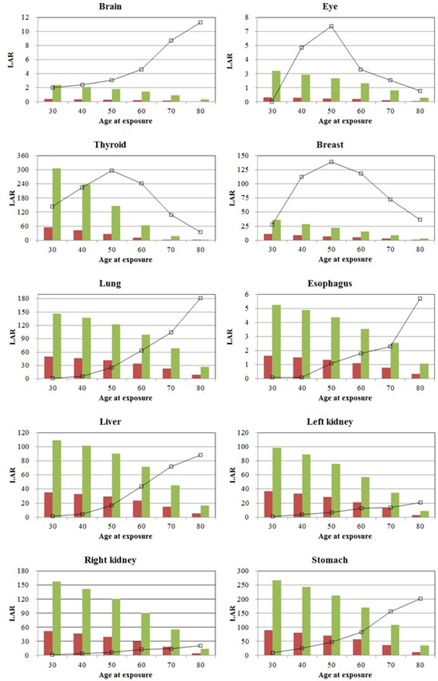
Risks of secondary cancer to organs resulting from external radiotherapy (green bars) and brachytherapy (red bars), as a function of age at exposure. The solid line in each graph shows baseline cancer risks in each organ.

During both brachytherapy and external radiotherapy, the right kidney was exposed to the highest dose (215.2 and 655.17 mGy, respectively) and the brain to the lowest dose (15.82 and 2.49 mGy, respectively) (Table 2). The differences between the lowest and highest doses were 86‐fold for brachytherapy and 41‐fold for external radiotherapy. The difference in ratio reflects differences in the two treatment modalities, in that brachytherapy primarily involves the impact of a low energy beam passing through the body, whereas external radiotherapy involves radiation scattered from the linac head.^29^, ^30^ Organ dose decreased in proportion to the distance of the organ from the target area. 3, 4 present the LARs associated with brachytherapy and external radiotherapy, respectively, depending on the age at exposure. All organs showed lower risks with increasing age at the onset of radiation exposure. Using an age at exposure of 30 years as a reference value, brachytherapy showed the highest LAR in the stomach (88.6) and the lowest LAR in the brain and eye (0.4), whereas external radiotherapy showed the highest LAR in the thyroid (305.1) and the lowest LAR in the brain (2.4). The thyroid was found to have the highest secondary cancer risk in external radiotherapy and the second highest secondary cancer risk in brachytherapy. Figure 4 shows histograms comparing LAR by organ for brachytherapy and external radiotherapy, as well as graphs showing the age‐specific cancer incidence of Korean women for these same organs, presented as baseline cancer risk for comparison purposes. Although LAR shows an inverse relationship to age at exposure, because radiation sensitivity is higher at a younger age, the age‐specific baseline cancer incidence of Korean women, especially for thyroid, eye, and breast cancers, tends to increase with increasing age. In contrast, the LAR for all other organs was higher than baseline cancer risks, showing that risks of secondary cancer were higher in women who received external radiotherapy and brachytherapy for cervical cancer.

**Table 2:** Physical doses to organs from external radiotherapy and brachytherapy (mGy) with one sigma uncertainty.

	*Brachytherapy*	*External Radiotherapy*
Brain	2.49±0.3	15.82±0.11
Eye	2.74±0.03	18.72±0.13
Thyroid	5.70±0.12	75.58±0.83
Breast	24.18±0.55	79.43±1.11
Lung	26.94±1.36	81.53±1.19
Esophagus	27.41±0.07	62.66±1.36
Liver	115.84±5.23	354.81±14.12
Left kidney	155.46±2.1	411.17±2.98
Right kidney	215.2±17.96	655.17±1.88
Stomach	76.71±1.6	230.96±1.12

**Table 3:** LAR for organs according to age at exposure to brachytherapy (per 100,000 population).

	*Age at Exposure*										
	*30*	*35*	*40*	*45*	*50*	*55*	*60*	*65*	*70*	*75*	*80*
Brain	0.4	0.4	0.3	0.3	0.3	0.3	0.2	0.2	0.1	0.1	0.1
Eye	0.4	0.4	0.4	0.3	0.3	0.3	0.2	0.2	0.2	0.1	0.1
Thyroid	54.4	48.8	42.3	34.8	26.1	18.3	11.3	6.1	3.1	1.3	0.4
Breast	11.5	10.4	9.3	8.2	7.1	6.0	4.9	3.9	2.9	1.9	1.0
Lung	49.8	48.2	46.4	44.2	41.5	38.1	33.8	28.8	23.1	16.3	9.0
Esophagus	1.6	1.6	1.5	1.5	1.4	1.2	1.1	0.9	0.8	0.6	0.3
Liver	35.5	34.4	33.0	31.5	29.4	26.8	23.4	19.4	14.8	10.2	5.5
Left kidney	37.2	35.6	33.6	31.3	28.6	25.4	21.5	17.2	13.2	8.4	3.4
Right kidney	51.5	49.3	46.6	43.3	39.6	35.2	29.7	23.8	18.2	11.6	4.6
Stomach	88.6	85.0	80.8	75.9	70.2	64.1	56.4	47.0	35.7	23.9	11.7

**Table 4:** LAR for organs according to age at exposure to external radiotherapy (per 100,000 population).

	*Age at Exposure*										
	*30*	*35*	*40*	*45*	*50*	*55*	*60*	*65*	*70*	*75*	*80*
Brain	2.4	2.3	2.1	2.0	1.8	1.6	1.4	1.2	0.9	0.6	0.3
Eye	2.8	2.7	2.5	2.3	2.2	1.9	1.7	1.4	1.1	0.8	0.4
Thyroid	305.1	273.6	237.0	194.9	146.3	102.4	63.1	34.4	17.1	7.2	2.4
Breast	35.9	32.4	29.0	25.5	22.1	18.8	15.4	12.2	9.0	6.0	3.1
Lung	146.8	142.1	136.9	130.5	122.4	112.4	99.6	84.9	68.2	48.2	26.5
Esophagus	5.2	5.1	4.9	4.7	4.3	4.0	3.5	3.0	2.6	1.8	1.1
Liver	108.7	105.2	101.2	96.4	90.0	82.2	71.6	59.5	45.4	31.2	16.7
Left kidney	98.5	94.2	89.0	82.8	75.7	67.2	56.8	45.5	34.8	22.2	8.9
Right kidney	156.9	150.2	141.8	132.0	120.6	107.1	90.6	72.5	55.5	35.3	14.1
Stomach	266.7	255.9	243.2	228.6	211.5	193.1	169.9	141.5	107.6	72.0	35.3

## IV. DISCUSSION

This study shows calculations of radiotherapy‐related secondary cancer risks in cervical cancer. External radiotherapy and brachytherapy are the radiotherapy modalities generally administered to women in our center with cervical cancer. Although treatment plans vary, depending on the phase of the disease and the condition of the patient, evaluations of radiotherapy‐induced risk assumed that patients undergo both external radiotherapy and brachytherapy. Brachytherapy is associated with numerous side effects, due to its high dose, although doses to other organs are affected by the attenuation of primary radiation. In external radiotherapy, however, doses to out‐of‐field organs are likely due to the significant amount of scatter radiation generated in the gantry head,^29^, ^30^ suggesting that significant amounts of radiation may reach nontarget organs. This study therefore evaluated the risks of low‐dose radiation exposure.

External radiotherapy involves a higher total dose than brachytherapy. Doses to organs decrease as the distance from the target area increases. Organ doses in brachytherapy were found to be higher to the esophagus than to the lungs, whereas organ doses in external radiotherapy were higher to the lungs. Relative to the lungs, doses to the thyroid were 4.72‐fold higher for brachytherapy, but only 1.07‐fold higher for external radiotherapy. This difference is attributable to the irradiation mechanism‐related differences in exposure levels between the two modalities. Irradiation of out‐of‐field organs during brachytherapy primarily results from the attenuation of primary radiation during its passage through the patient's body, whereas irradiation during external radiotherapy usually results from scattered radiation in the gantry head, leading to higher irradiation levels delivered to organs positioned in the periphery during external radiotherapy than during brachytherapy. Despite its large distance from the target area, secondary cancer risk to the thyroid was greater than to other organs, primarily because the thyroid has higher parameters for cancer risks. The risk of thyroid cancer, however, tends to decrease with age. The high level of secondary cancer risk relative to radiation dose indicates the need for post‐treatment follow‐up measures or the use of dose delivery technologies that minimize the dose as much as possible.

## V. CONCLUSIONS

This study measured radiation doses to external organs resulting from the treatment of cervical cancer with brachytherapy and external beam radiation, and calculated the incidence of secondary cancer resulting from radiation exposure of normal organs. Radiotherapy is a widely employed first‐line treatment for cervical cancer. Due to the decreasing age of patients at disease onset and their increasing life expectancy, this study was designed to estimate the impact of radiotherapy on post‐treatment secondary cancer risk. While all investigated organs showed some levels of risk, these risks were especially pronounced in the liver, kidney, and stomach. These results suggest the need for a follow‐up study to establish a database on secondary cancer risks related to radiotherapy in patients with cervical cancer, and to explore strategies to minimize the impact of radiotherapy on risks of secondary cancer.

## ACKNOWLEDGMENTS

This work was supported by the National Nuclear R&D Program through the National Research Foundation of Korea (NRF), funded by the Ministry of Education, Science and Technology (NRF‐2015M2A2A7A02045273).

## COPYRIGHT

This work is licensed under a Creative Commons Attribution 3.0 Unported License.
